# Sclerobanding Is a Novel Technique for the Treatment of Second- and Third-Degree Hemorrhoidal Disease. Reply to Jongen et al. Comment on “Pata et al. Sclerobanding (Combined Rubber Band Ligation with 3% Polidocanol Foam Sclerotherapy) for the Treatment of Second- and Third-Degree Hemorrhoidal Disease: Feasibility and Short-Term Outcomes. *J. Clin. Med*. 2022, *11*, 218”

**DOI:** 10.3390/jcm11113078

**Published:** 2022-05-30

**Authors:** Francesco Pata, Luigi Maria Bracchitta, Giancarlo D’Ambrosio, Salvatore Bracchitta

**Affiliations:** 1 Department of Surgery, Nicola Giannettasio Hospital, 87064 Corigliano-Rossano, Italy; 2 La Sapienza University, 00185 Rome, Italy; 3 Primary Care Department, ATS Città Metropolitana, 20100 Milan, Italy; luigimaria.bracchitta@gmail.com; 4 Department of General Surgery, Surgical Specialties and Organ Transplantation, La Sapienza University, 00161 Rome, Italy; giancarlo.dambrosio@uniroma1.it; 5 Coloproctology Centre, Clinica del Mediterraneo, 97100 Ragusa, Italy; dott.salvatorebracchitta@gmail.com

We thank Johannes Jongen and colleagues for their correspondence [1] about our article [2], which gives us the opportunity to better clarify the peculiar features of Sclerobanding in the treatment of hemorrhoidal disease (HD).

The key elements on which any surgical or office-based technique for HD are based (ligation, injection and excision) have already been historically described [3]. Thus, what makes a technique original is the novelty of the device, the agent used and/or the distinctive features of the procedural steps.

Dr Jongen points out that five studies, which we mentioned in the discussion of our article, have already reported the concomitant use of rubber band ligation (RBL) and sclerotherapy (SCL). However, as we already highlight in our article, “concomitant” does not imply “combined”. Two of these studies reported the selective use of RBL or Sclerotherapy in different nodules in the same patient [4,5]; another study described the use of sclerotherapy as the first step to reduce the volume of the hemorrhoidal nodule which is then treated by RBL [6]. Regarding the two oldest studies, one reported a technique with unclear steps [7], and another mentioned a “modified” RBL as a part of the procedure [8]. Moreover, none these studies have reported the use of 3% polidocanol foam as a sclerotizing agent, which presents additional features of effectiveness and safety and represents an indispensable component of our technique.

Sclerobanding is a standardized procedure based on RBL of the hemorrhoidal nodule above the dentate line, followed by the injection of 3% polidocanol foam above the band in the same nodule to obtain a synergic rather than an additive effect: on the one hand, the 3% polidocanol injection may prevent the early slippage of the rubber band, the risk of delayed bleeding, increasing the fibrosis in the ligated site, with an enhanced “lifting” effect; on the other hand, the rubber band may avoid the spreading of polidocanol foam in the surrounding tissues, reducing some significant complications, especially in the anterior area (abscess, acute prostatitis, sepsis) [9]. To the best of our knowledge, no other authors have described a procedure with all these features before. This demonstrates the novelty of Sclerobanding in comparison with similar procedures, although born by two distinguished techniques already in use among coloproctologists.
